# Barriers and Facilitators of Physical Activity Participation among Children and Adolescents with Intellectual Disabilities: A Scoping Review

**DOI:** 10.3390/healthcare10020233

**Published:** 2022-01-26

**Authors:** Siyi Yu, Taijin Wang, Tianwei Zhong, Yingtao Qian, Jing Qi

**Affiliations:** College of Physical Education and Health Sciences, Zhejiang Normal University, Jinhua 321004, China; Yusiyi@zjnu.edu.cn (S.Y.); Wangtaijin@zjnu.edu.cn (T.W.); zhongtianwei@zjnu.edu.cn (T.Z.); qianyingtao@zjnu.edu.cn (Y.Q.)

**Keywords:** children and adolescents, intellectual disability, physical activity, barriers, facilitators, scoping review

## Abstract

*Background:* Children and adolescents with intellectual disabilities (ID) have low levels of physical activity (PA). Understanding factors influencing the PA participation of this population is essential to the design of effective interventions. The purposes of this study were to identify and map the barriers and facilitators of PA participation among children and adolescents with ID. *Methods:* A scoping review was conducted in accordance with established methodology. Articles were evaluated for relevance using predetermined inclusion criteria in eight databases. Extracted barriers and facilitators were classified using the social ecological model as individual, interpersonal, or environmental factors. *Results:* Thirty-two studies published between 1992 and 2020 were included (24 quantitative, 6 qualitative, and 2 mixed-method). Thirty-four factors were identified. The most commonly reported barriers included disability-specific factors, low self-efficacy, lack of parental support, inadequate or inaccessible facilities, and lack of appropriate programs. The most commonly reported facilitators included high self-efficacy, enjoyment of PA, sufficient parental support, social interaction with peers, attending school physical education (PE) classes, and adapted PA programs. *Conclusions:* Continued exploration of factors influencing PA participation is required among children and adolescents with ID. Future interventions should involve families, schools, and wider support network in promoting their PA participation together.

## 1. Introduction

Physical activity (PA) is defined as any bodily movement produced by skeletal muscles that requires energy expenditure [1] and is characterized by its modality, frequency, intensity, duration, and context of practice [2]. PA promotes numerous physical and mental health benefits in children, including children and adolescents with disabilities [3,4,5]. Regular and adequate levels of PA can improve children’s cardiorespiratory and muscular fitness, bone health, and cardiovascular and metabolic health biomarkers, reduce symptoms of anxiety and depression, and help to maintain a healthy weight [6]. Despite the physiological and psychological health benefits associated with PA participation, previous studies reported that children with intellectual disabilities (ID) did not meet the PA guideline of at least 60 min of moderate-to-vigorous-intensity physical activity (MVPA) per day [7,8,9]. In addition, children and adolescents with ID are less active than their counterparts without disabilities [10,11].

ID is characterized by significant limitations in intellectual functioning and adaptive behavior, which covers many everyday social and practical skills and originates before the age of 18 years [12]. Children and adolescents with ID tend to have greater sedentary behaviors because associated physical, sensory, and/or cognitive impairments place them at a disadvantage when participating in sports and games with typical peers [13]. Moreover, environmental barriers exist, such as limited access to facilities and recreation areas, inaccessible sports and fitness equipment, and little knowledge of staff in adapting games and sports for children with disability, resulting in their limited PA participation [14]. Thus, this population is at greater risk of low levels of PA and increased rates of overweight, obesity, and chronic health conditions [14,15,16,17]. Increasing levels of PA could therefore be effective in improving relevant health outcomes for this population [18]. Understanding the barriers and facilitators of PA participation among children and adolescents with ID is fundamental to the design of effective interventions in this group.

Many studies have examined factors impeding or facilitating PA participation among children and adolescents with ID. To date, two recent systematic reviews [19,20] synthesized the correlates of PA in children and adolescents with ID. One included 15 related studies published in the past 10 years and identified 48 individual correlates that were predominantly focused on intrapersonal level, such as motor development, age, and cardiorespiratory fitness [19]. The other work included 10 studies and summarized 29 correlates at intrapersonal level, social level, and physical environmental level [20]. These systematic reviews mainly included cross-sectional research design studies but did not discuss identified factors influencing the PA of children and adolescents with ID from interventional or qualitative studies. We did not find any reviews synthesizing literature on barriers to PA and facilitators specifically targeting PA behaviors among children and adolescents within the ID group. Therefore, the purpose of this scoping review is to map the existing literature on the barriers and facilitators of PA participation among children and adolescents with ID in terms of volume, nature, and characteristics of primary research [21]. The findings may provide information for intervention design and future research directions. In this review, barriers to PA were defined as any physiological, psychological, or socio-ecological conditions reported to reduce or negatively affect a person’s participation in PA [22]. Facilitators of PA were defined as programs, interventions, or factors that may improve PA participation [23]. Facilitators were differentiated from preferences which were defined as characteristics or features of exercise, PA, or an exercise program, in particular one that participants identified as enjoyable and were excluded from [22].

## 2. Methods

This study adopted an established six-stage scoping review protocol proposed by Arksey and O’Malley [21] and further refined by Levac et al. [24] and followed the Preferred Reporting Items for Systematic Review and Meta-Analysis Extension for Scoping Reviews (PRISMA-ScR) statement [25]. The study protocol was registered with the Open Science Framework (https://osf.io/kp5cd (accessed on 20 November 2021)).

### 2.1. Stage 1: Identify the Research Question

The primary question to be addressed through this scoping review was “What are the barriers to PA and facilitators of PA participation among children and adolescents with ID?”

### 2.2. Stage 2: Identify Relevant Studies

A comprehensive search was performed using eight electronic databases: Web of Science (WOS), Academic Search Premier (ASP), MEDLINE, Education Source (ES), Education Resource Information Center (ERIC), PsycINFO, Psychology and Behavioral Sciences Collection, and Scopus. The search gave access to a range of health, sport, psychology, medicine, and education journals. A list of keywords and alternative keywords were created, combined using Boolean operators (“AND”, “OR”), and included in the aforementioned search databases. The English search strings included:

1. “physical activit *” OR “PA” OR “MVPA” OR exercise * OR “health behavior” OR “motor activit *” OR “sport *” OR “physical education” AND
2. Youth * OR “young athlete *” OR adolescent * OR teenager * OR child * OR childhood OR student * AND
3. “intellectual disability *” OR “mental retardation” OR “intellectual development” OR “developmental disability *” AND
4. Correlate * OR factor * OR reason * OR predictor * OR barrier * OR facilitator *

Articles published between 1950 and 2020 were eligible for review. All articles were published in English and available in full-text format. Hand searching and snowballing techniques from the reference lists of systematic reviews and key references were performed to identify potentially relevant studies not captured by database searches [26].

### 2.3. Stage 3: Study Selection

The inclusion criteria were as follows: (1) empirical research (qualitative and quantitative) that focused on identifying barriers and facilitators related to PA among children and adolescents with ID. All reported dimensions of PA were eligible for inclusion, for example, mode, frequency, duration, and intensity of PA [27]. PA intensities are categorized as follows: light PA (LPA: 1.6–2.9 metabolic equivalents of task (METs)), moderate PA (MPA: 3.0–5.9 METs), vigorous PA (VPA: ≥6.0 METs), and MVPA (≥3.0 METs) [27]; (2) the study participants included were children and adolescents with ID (age range 5–17 years) or parents and/or caregivers giving information regarding their children with ID; (3) full-text publication in English; and (4) published in a peer-reviewed journal before 31 December 2020.

The exclusion criteria were as follows: (1) studies in which PA and its barriers or facilitators were not the main outcome. (2) more than 50% of the participating children did not have ID, and the results were not presented separately; (3) studies were not empirical (e.g., conceptual, review, or philosophical only), and (4) editorials without extensive references, dissertations, theses, conference proceedings, and abstracts. This review included only original, peer-reviewed published articles and did not include any grey literature due to limited time and resource and language barriers.

All references were exported to EndNote Online, and duplicates were identified and removed through the software and manual review of the citation list. Articles were assessed for eligibility by title and abstract first, according to the inclusion criteria. Full-text articles were obtained where studies met criteria or could not be excluded on the basis of title and abstract alone. The identified studies were determined by four reviewers (S.-Y.Y., T.-J.W., T.-W.Z., and Y.-T.Q.) independently according to the inclusion and exclusion criteria. A fifth reviewer (J.Q.) was consulted in case of disagreement. Eligible studies were those that reported barriers and/or facilitators of engaging in PA using quantitative, qualitative, or mixed methods.

### 2.4. Stage 4: Charting the Data

Data were extracted and presented according to the methodology of the included studies. The identifiers and variables included first author and year of publication, type of study, geographic location, sampling strategy, participant details (i.e., sample size, age, gender, and ID level), theory, research design, measures, and dimensions of PA.

### 2.5. Stage 5: Collating, Summarizing, and Reporting Results

The data collected from the identified studies were inputted into a table and were analyzed descriptively. The social ecological model is a framework that aims to understand multiple levels of influence on specific health behaviors, including intrapersonal (individual), interpersonal, organizational, community, physical environmental, and policy [28]. This model leads to the explicit consideration of multiple levels of influence that broadens options for interventions [28]. Previous research [29,30,31] also demonstrated that this framework is useful in trying to understand facilitators and barriers influencing PA behavior in vulnerable populations. Thus, it was used as the theoretical framework for helping categorize factors and interpret our findings. Aligned with the guidelines of a scoping review, none of the studies were evaluated for quality and all works reported in this review were based on direct presentation of results from the authors of the included studies [21].

### 2.6. Stage 6: Consulting with the Experts

We aimed to enhance the methodological rigor of this review through expert consultation to gain additional sources of information and perspectives [21,24]. Several researchers (professional stakeholders) with expertise in PA participation of children and adolescents were involved throughout this review process. The experts were invited to review and offer suggestions for the searching strategies. According to the results of expert consultation, we optimized the key terms (i.e., added terms “physical education” and “young athlete *”, changed term “sport activit *” to “sport *”), and refined the inclusion and exclusion criteria of studies (e.g., identified specific types of studies or articles to exclude). Following the search and summary of the literature, the experts also provided perspectives on our thematic synthesis to help us finalize the themes and interpret of the findings.

## 3. Results

### 3.1. Searching Results

**Figure 1:**
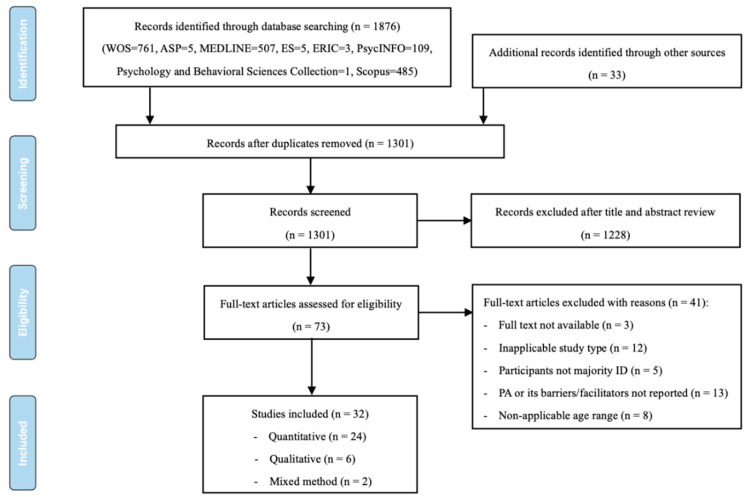
Flowchart of search and study selection.

The initial search identified 1876 studies (WOS, *n* = 761; ASP, *n* = 5; MEDLINE, *n* = 507; ES, *n* = 5; ERIC, *n* = 3; PsycINFO, *n* = 109; Psychology and Behavioral Sciences Collection, *n* = 1; Scopus, *n* = 485). Thirty-three additional studies were identified through related reviews. After removing duplicates from the original sample (*n* = 1909), title and abstract screening of 1301 articles was performed, from which 1228 studies were excluded. The researchers read the full text of the remaining 73 articles and excluded another 41. Finally, 32 studies were included in this review. Figure 1, adapted from the PRISMA group [32], displays the detailed search and study selection process.

### 3.2. Study Characteristics

Table 1 summarizes the details of the studies that met the inclusion criteria. The final 32 articles selected for review were published between 1992 and 2020, 24 of which (75%) were published after 2010. These studies we conducted in the USA (12), UK (5), Canada (3), China (3), Australia (2), Iceland (1), Italy (1), the Netherlands (1), the Philippines (1), Saudi Arabia (1), Spain (1), and Trinidad and Tobago (1). A total of 24 studies employed quantitative methods of data collection, 6 used qualitative data collection methods, and the 2 remaining studies adopted mixed methods. Of the quantitative and mixed-method studies, 18 articles employed a cross-sectional design, 6 adopted intervention, one used a longitudinal design, and one utilized a case design. The six qualitative studies all employed a phenomenological design. Of the quantitative studies, 17 studies used objective measures including accelerometers (*n* = 11), pedometers (*n* = 3), heart rate monitors (*n* = 4), and quantitative observation (*n* = 6) to quantify PA. Nine studies used questionnaires as subjective measures. Two of the quantitative studies utilized more than one measurement tool. The intensity and duration of PA were presented as different ways due to different measurements. Among included quantitative studies, 17 studies used different PA dimensions including LPA, MPA, MVPA, and number of steps per day. Another 9 studies used regular PA, PA frequency, and PA perceptual characteristics based on subjective PA questionnaires. The qualitative studies used interviews (*n* = 4) and focus groups (*n* = 2) to explore the barriers and facilitators to PA among children and adolescents with ID. The mixed-method studies involved objective (e.g., accelerometers, quantitative observation, heart rate monitors) and subjective measurements (e.g., questionnaire, interviews). These two studies used different dimensions including MPA and MVPA. Of the 32 studies, 15 studies used a purposive sampling strategy, 10 studies used a convenience sampling strategy, and 7 studies did not provide an indication of the sampling strategy. The sample size ranged from 3 to 535, including one with more than 500 participants, 4 with 100 to 500 participants, 16 with 30 to 100 participants, and 11 with less than 30 participants. In all, 6 (19%) stated the use of theories, including social cognitive theory (*n* = 2), self-determination theory (*n* = 2), occupational perspective theory (*n* = 1), and dynamic systems theory (*n* = 1).

### 3.3. Thematic Synthesis

The barriers and facilitators of PA participation among children and adolescents with ID are classified into three groups of studies using different research methods. Specifically, barriers and facilitators are presented under individual, interpersonal, and environmental levels of influence based on the social ecological model [28] (Table 2).

#### 3.3.1. Barriers to Participating in PA

##### Qualitative Studies

The included qualitative studies identified barriers to PA participation among children and adolescents with ID based on the perceptions of parents, teachers, and adolescents with ID. Any dimension of PA was not available in these studies. At the individual level, the results of studies showed that conditions associated with ID, such as developmental delays [57], ear problems [49], and common characteristics associated with DS (including hypotonia, congenital heart defects, and communication impairments) [51] were identified as physiological factors that inhibited PA participation in children and adolescents with ID. Low self-efficacy [38] and lack of understanding on the importance of PA and its benefits for health [48] were identified as cognitive and psychological barriers to PA participation. Interpersonal factors are related to interpersonal processes and primary groups, such as family and peers [63], influencing PA participation among children and adolescents with ID. Lack of parental support (including lack of parents’ company [46,51], lack of family’s financial support [46,57], lack of transport support [49], lack of information for parents on how to conduct home-based activities [57]), and parent’s vigilance and overprotection [46,51] were identified as family barriers to PA participation among children and adolescents with ID. In addition, lack of social networks (e.g., lack of social connectedness with others) was also identified as an interpersonal barrier to PA participation among children and adolescents with ID [38]. At the environmental level, inadequate or inaccessible facilities [46] and lack of appropriate programs [51,57] were identified as social environmental barriers to PA participation among children and adolescents with ID. Poor weather, as one of the natural factors, prevented this population from participating in outdoor activity and thus decreased their PA [38,46].

##### Quantitative Studies

At the individual level, low motor development (e.g., low locomotor and object control skills) [9,33,45] was identified as a barrier that influenced MVPA or the number of steps per day among children and adolescents with ID. Low self-efficacy [43] and a preference for indoor activities [62] were identified as cognitive and psychological barriers that influenced regular PA and rating perceived exertion of PA participation among children and adolescents with ID. At the interpersonal level, lack of a social network (e.g., have fewer friends) was identified as a barrier that influenced regular PA among children and adolescents with ID [43]. At the environmental level, teacher and classroom-related factors were examined in previous studies. The results of the study found that lesson contexts organized by PE teachers (e.g., allocating the substantial amount of lesson time for management) and teaching behaviors (e.g., spending considerably more time transmitting physical education (PE) knowledge), which reduced opportunities for students to participate in MVPA, were identified as barriers [56].

##### Mixed-Method Studies

Lack of parental support [59] and lack of public transportation [59] were, respectively, identified as barriers at the interpersonal and environmental levels that influence MPA among children and adolescents with ID in one study using mixed methods.

**Table 2:** Barriers and facilitators identified of PA participation among children and adolescents with ID.

**Themes**	**Dimensions of PA**							
	**Intensities of PA**			**Steps**	**Subjective PA Questionnaires**			**N/A**
	**LPA**	**MPA**	**MVPA**	**Steps/Day** **-Average Daily Steps Counts**	**Regular PA (Yes or No)**	**PA Frequency (Times Per Week)**	**PA Perceptual** **Characteristics (Perceived** **Exertion)**	
**Barriers**								
**Individual factors**								
**- Physiological factors**								
Conditions associated with ID								[49,51,57]
**- Motor development**								
Low motor development			[9]	[33,45]				
**- Cognitive and psychological factors**								
Low self-efficacy					[43]			[38]
Lack of understanding about importance of PA and its benefits to health								[48]
Preference for indoor activities							[62]	
**Interpersonal factors**								
**- Family**								
Lack of parental support		[59]						[46,49,51,57]
Parents’ vigilance and overprotection								[46,51]
**- Social network**								
Lack of social network					[43]			[38]
**Environmental factors**								
**- Social environment**								
Inadequate or inaccessible facilities								[46]
Lack of appropriate programs								[51,57]
Lack of public transportation		[59]						
**- School environment**								
Lesson contexts (management)			[56]					
Teaching behaviors (transmit knowledge)			[56]					
**- Natural environment**								
Poor weather								[38,46]
**Facilitators**								
**Individual factors**								
**- Physical abilities**								
Physical skills			[53]					[51]
**- Cognitive and psychological factors**								
High self-efficacy					[43]			[38]
Weight loss						[40]		
Enjoyment of PA	[44]	[44]			[43]			[48,49]
Personality traits								[51]
Caregiver’s high educational level					[54]			
**Interpersonal factors**								
**- Family**								
Sufficient parental support						[37]		[38,48,49,51,57]
Positive parental beliefs						[41]		
Positive role of siblings								[51,57]
**- Social network**								
Positive social interaction with peers						[11,52]		[38,49,51,57]
Positive coach–athlete relationship						[37]		
**Environmental factors**								
**- Social environment**								
An exergaming context			[34]					
Adequate and available resources						[37]		
Adapted PA programs	[50]		[39,53]					[51]
**- School environment**								
Attending PE classes and participating PA during recess			[40,47,55,58,60,61]	[42,45]				[38]
Inclusive PE programs	[35]						[35]	
High autonomy–supportive climates on PA			[36]					
Lesson contexts (skill practice)			[56]					
Teaching methods								[48]
A strong home-school link								[48]

ID, intellectual disabilities; LPA, light physical activity; MPA, moderate physical activity; MVPA, moderate to vigorous physical activity; N/A, not available; PA, physical activity; PE, physical education.

#### 3.3.2. Facilitators of Participating in PA

##### Qualitative Studies

Facilitators of PA participation among children and adolescents with ID reported by the included qualitative studies were also identified from perceptions of parents, teachers, and adolescents with ID. At the individual level, physical skills were identified as facilitators of participating in PA among children and adolescents with ID [51]. Cognitive and psychological factors, such as high self-efficacy [38], enjoyment of PA [48,49], and personality traits (e.g., enthusiastic and determined) [51] were also facilitators. At the interpersonal level, sufficient parental support (e.g., parents’ positive role model, parental company and logistic supports) [38,48,49,51,57], positive role of siblings [51,57], and positive social interactions with peers [38,49,51,57] were identified as facilitators of participating in PA among children and adolescents with ID. At the environmental level, PA programs available in the community adapted for children and adolescents with ID were identified as social environment facilitators of participating in PA among children and adolescents with ID [51]. Attending PE classes [38], teaching methods, and a strong home-school link [48] were identified as school environment factors of participating in PA among children and adolescents with ID.

##### Quantitative Studies

At the individual level, physical skills (e.g., riding a bicycle) were identified as physical ability factors that influence MVPA among children and adolescents with ID [53]. Wanting to lose weight [40], high self-efficacy [43], and enjoyment of PA [43,44] were identified as cognitive and psychological facilitators that influence PA frequency, regular PA, LPA, and MPA among children and adolescents with ID. In addition, caregiver’s higher educational level was another individual facilitator that influenced regular PA among children and adolescents with ID [54]. At the interpersonal level, sufficient parental support (e.g., parents’ company) [37] and positive parental beliefs of the benefits of PA for their child [41] were identified as family factors that influence PA frequency among children and adolescents with ID. In addition, positive social interactions with peers [11,52] and positive relationships with the coach [37] were identified as social network facilitators that influence PA frequency among children and adolescents with ID. At the environmental level, an exergaming context implemented at home or at school was identified as a facilitator that influenced MVPA among children and adolescents with ID [34]. Adequacy and availability of environmental resources (e.g., access to transportation) were identified as social environment factors that influenced PA frequency among children and adolescents with ID [37]. PA programs available in the community adapted for children and adolescents with ID were also identified as facilitators that influenced LPA and MVPA among children and adolescents with ID [50,53]. In terms of school factors, attending PE classes and participating in physical activities during school recess [40,42,45,47,55,58,60,61] were identified as key facilitators that influenced MVPA or number of steps per day among children and adolescents with ID. Inclusive PE programs (e.g., a peer-tutored PE program) [35] were also identified as school facilitators that influenced the LPA and PA frequency of children and adolescents with ID. In addition, high autonomy-supportive instructional climates [36] and PE lesson contexts focused on skill practice [56] were identified as facilitators that influenced MVPA among children and adolescents with ID.

##### Mixed-Method Studies

An adapted PA program using group video conferencing for the promotion of PA [39] was identified as a facilitator that influenced MVPA among children and adolescents with ID at the environmental level.

## 4. Discussion

This scoping review provided an overview of the barriers and facilitators to PA participation among children and adolescents with ID. A systematic search yielded 32 studies published between 1992 and 2020. Research has steadily increased in this area over the last few years, which showed that scholars have paid increasing attention in the PA of children and adolescents with ID in the past decade. Among the included studies, quantitative studies are the most numerous. Most of these studies had problems related to the cross-sectional design and the sample (e.g., small sample size). The cross-sectional research design cannot indicate causality. The results cannot be generalized because of the relatively small sample size. Thus, more longitudinal studies are needed to identify factors that have causal associations with PA [64]. Further studies in larger samples are also necessary to improve the study quality and generalizability of findings [65]. There was a dearth of experimental studies using random assignment or that were well-controlled investigations with contrasting groups or conditions. Randomized control trials/quasi-experimental studies are useful to establish the efficacy of interventions targeting barriers to PA, which is important and necessary for effectively promoting PA participation among children and adolescents with ID [65,66]. Studies using this design must address quality control in design and reporting to ensure the usability of findings [67].

The results of the quantitative and mixed-method studies included in this review showed that different barriers and facilitators could influence different dimensions of PA in children and adolescents with ID. However, the evidence available based on these studies was limited and incomplete. It would therefore be a research direction to comprehensively examine the relationships between barriers or facilitators and various dimensions of PA (e.g., different intensities, frequencies, and modes) in this ID groups.

Qualitative studies help to explore and understand full-breadth issues in relation to the PA participation experienced by a specific population [23]. Therefore, it would be best suited to the profound exploration of the specific barriers and facilitators of PA participation among children and adolescents with ID [68]. However, only 19% (*n* = 6) of the studies included in this review employed a qualitative research design. Qualitative studies are needed to address how children and adolescents with ID participate in PA and why their PA levels are lower than their peers without disabilities [47,69]. Theoretical frameworks were designed to help comprehensively understand the relationship between factors and the mechanisms by which they affect behavior [22]. However, only 19% (*n* = 6) of the research used a theoretical framework to guide their studies. Studies using the behavioral theoretical frameworks are urgently needed to better understand healthy behavioral patterns and guide the development of effective interventions to promote PA among children and adolescents with ID [22,66].

Based on the social ecological model, our synthesis of the studies identified 34 factors primarily related to individual, interpersonal, and environmental elements at several levels of influence. The most predominant barriers identified in this review at the individual level were disability-specific factors, including conditions associated with ID (e.g., developmental delays, ear problems, communication impairments) and low motor development. This finding is consistent with previous reviews on examining parental perceptions of facilitators and barriers to PA for children with ID [70]. Children’s conditions associated with ID may decrease the activity levels because of their influence on body structure and function [51]. This finding suggests a need for greater emphasis on home- and community-based programs that promote health wellness issues for this population to help understand the physical limitations that they may present, make appropriate adaptations to PA, and provide them with opportunities for PA participation [46,57]. The low motor development of children and adolescents with ID relates to their generally slow developing of basic physical skills required to be active, and increased motor development has been identified as an underlying mechanism to promote PA participation [9,19,71]. Thus, consideration of how to improve the physical fitness and motor skills of children and adolescents with ID may have a long-term influence on the amount of PA they undertake. Previous studies confirmed that motor skill interventions had positive effects on improving the motor development of children and adolescents with ID [72,73]. Therefore, professionals are recommended to develop more effective motor skill interventions, such as developmental physical education programs, therapeutic sensorimotor training, or intensive motor skill training, to increase their motor development [74].

Self-efficacy was the second most frequently reported factor influencing the PA participation of children and adolescents with ID at the individual level. High self-efficacy can increase the intrinsic motivation to participate in PA among children and adolescents with ID, whilst low self-efficacy thwarted intrinsic motivation, highlighting the importance of considering their self-efficacy in activities when attempting to encourage PA in this population [38]. Parents, PE teachers, and researchers should be aware that the activities must be tailored to the individual in relation to their self-efficacy and provide social support in activities to increase their self-efficacy to maintain interest and enjoyment, instead of just promoting activities. In addition, the perceptions and attitudes of participating in the PA of children and adolescents with ID were identified as individual factors influencing their PA participation. For example, lack of understanding on the importance of PA and its benefits to health may inhibit their PA, while enjoyment of PA and wanting to lose weight may promote their PA behavior. Therefore, providing children and adolescents with ID a variety of opportunities to successfully participate in PA may be a logical first step toward increasing enjoyment; this, in turn, could lead to PA becoming a preferred activity [43]. Moreover, there is a need to develop a multimodal intervention that combines PA and health education to further educate them concerning the knowledge and benefits of PA to improve their cognition and promote their positive attitudes toward PA [49].

Parent-related factors were the most frequently reported factor influencing the PA participation of children and adolescents with ID at the interpersonal level. In particular, parental support, as both a barrier and a facilitator, was concluded to be the prominent concern. Sufficient parental support, including parents’ positive models, company and supports (e.g., transportation supports, financial supports, providing encouragement) facilitated the PA participation of children and adolescents with ID [48,49,57]. By contrast, lack of parental support and parents’ lack of professional knowledge related to PA inhibited the PA participation of their children with ID [38,51,57]. Therefore, being positive role models, supplying company and encouragement, and providing transport and financial support may be the integral ‘gatekeeper’ roles that parents play in promoting the PA participation of children and adolescents with ID [70,75]. In addition, parents’ high levels of overprotection and concerns relating to their child’s competence for participating in various physical activities may prevent their child from conducting healthy and helpful physical activities [46,51]. Parents are recommended to learn about related PA guidelines and the safe physical activities available to and appropriate for their children with ID [46]. These findings suggest that greater professional support and advice related to PA participation among children and adolescents with ID need to be offered to parents; in turn, parents could then provide more supports and encouragement for their children.

Social networks were also identified as a key factor influencing the PA participation of children and adolescents with ID at the interpersonal level. The results of this review showed that social connectedness, teamwork, and competition in sporting activities with friends facilitated intrinsic motivation to participate in PA in this population, while a lack of social connectedness leads to feelings of alienation and inactivity [38]. Parents, teachers, and program planners should aim to promote and encourage a social element as a reason to participate in activities, and support children and adolescents with ID to socially interact with peers with and without disabilities to encourage them to participate more in PA [38,75].

PE class-related factors were the most frequently reported factor influencing the PA participation of children and adolescents with ID at the environmental level. This result confirmed the Ecological models of health behavior that emphasize the environmental contexts of behavior [28]. The population of children and adolescents with ID depends more on schools to accumulate their PA, especially the school PE curriculum [38,42]. Previous studies proved that PE lesson contexts focused on skill practice and that high autonomy–supportive instructional climates promoted the PA participation of children and adolescents with ID [36,56]. Therefore, during school PE classes, the PE teachers are recommended to make appropriate adaptations to PA programs and choose activities focusing on games and skills to increase the frequency and intensity of activities to make ID students participate in adequate PA [47,58]. They are also recommended to design adapted interventions such as peer-tutored PE programs or group-based activities that can offer the potential for social interaction and support to encourage the students to participate more activities in PE classes [35,39]. However, studies using randomized control trials to examine the effects of the teaching methods of activities on promoting PA during PE classes are still needed.

Environmental facilities and resources and PA programs adapted for children and adolescents with ID available in the communities were also two identified key factors influencing the PA participation of children and adolescents with ID at the environmental level. They were both barriers and facilitators, depending on their adequacy or lack thereof. Adequate and accessible facilities and resources provide a basic guarantee for PA participation of children and adolescents with ID. As part of objective social support, they may improve enjoyment and motivation towards PA, hence increasing the likelihood of PA behavior change [23,76]. Local governments and community organizations should consider improving the accessibilities of facilities or resources in communities, and make efforts to provide more PA programs adapted for children with disabilities, to support the extensive PA participation of children and adolescents with ID [77].

Poor weather was frequently identified as a barrier to the PA participation of children and adolescents with ID at the environmental level. On the one hand, self-efficacy should be strengthened in order for the child to be able to make more effort and overcome difficulties and continue participating in PA in special situations (e.g., poor weather conditions) [76,78]. On the other hand, more indoor-based physical activities should be introduced by parents or schools to replace outdoor activities that are less suitable in real ‘bad’ weather [49].

The strengths of this paper include the use of a systematic search strategy to conduct a scoping review and produce an extensive yield of relevant literature and its inclusivity of a range of study designs, allowing us to provide a more comprehensive overview of the evidence base. Furthermore, the use of the social ecological model allowed the researchers to explore the multiple influences on PA at different levels. It provided a framework to categorize factors and to highlight where previous research focused and what future research directions are required [19].

Some limitations should also be considered when interpreting the results of this review. Due to the nature of scoping reviews, we did not assess the quality of the included studies, which may have influenced the quality of the results of the studies. This may be a perceived limitation of our methodological frameworks. If possible, it is recommended that quality assessment using validated instruments should be factored into the framework of scoping reviews and add the criteria to the selection of studies to be charted in future research [79]. Language bias may be present because studies that were not written in English were excluded. Finally, the relative importance of each factor should be considered, because the strength of the factor is mostly unclear, and which factor is the most important is uncertain [78]. Therefore, some of the findings should be interpreted with caution. Moreover, issues relevant to the potential limitations of social ecological models need to be considered. The social ecological model has a lack of sufficient specificity to guide conceptualization of a specific problem, identification of appropriate interventions, or clarity in determining when and where to intervene [63]. Given this potential for lack of specificity, theoretical and conceptual development is essential to the advancement of social ecological models to guide the identification of PA factors and target PA promotion among children and adolescents with ID.

## 5. Conclusions

This study conducts a scoping review to identify barriers and facilitators of PA participation among children and adolescents with ID based on a social ecological model. The results indicated that disability-specific factors, low self-efficacy, lack of parental support, inadequate or inaccessible facilities, and lack of appropriate programs were the most commonly reported barriers. High self-efficacy, enjoyment of PA, sufficient parental support, social interaction with peers, attending school PE classes, and adapted PA programs were the most commonly reported facilitators. Given the findings from this scoping review, there is a need for continued exploration of the barriers and facilitators of PA participation among children and adolescents with ID by more qualitative, longitudinal, and interventional studies. By understanding the relationships between barriers and facilitators and the different dimensions of PA, interventions can be better designed and adapted to encourage greater PA participation for children and adolescents. Such work may be vital to improve this population’s health and growth.

## Figures and Tables

**Table 1:** Descriptive statistics of included studies.

First Author (Year)	Type of Study	Geographic Location	Sampling Strategy	Participant Details				Theory	**Research** **Design**	**Measures**
				Sample Size	Age	Gender	ID Level			
Alhusaini (2020) [33]	Quantitative	Saudi Arabia	purposive	78 (37DS/41TD)	8–12	male	DS	n/a	cross-sectional	pedometer
Pincus (2019) [34]	Quantitative	USA	purposive	3	16–18	1 male 2 female	moderate sever unspecified	n/a	intervention	quantitative observation (OSRAC-H)
Wouters (2019) [9]	Quantitative	Netherlands	purposive	68	2–18	43 male 25 female	moderate to severe	n/a	cross-sectional	accelerometer
Gobbi (2018) [35]	Quantitative	Italy	convenience	19	17.4 ± 1.7	15 male 4 female	mild to moderate	n/a	case study	accelerometer questionnaire
Johnson (2018) [36]	Quantitative	USA	could not be determined	32 (14DD/18TD)	5–9 (6.89 ± 1.11)	9/11 male 5/7 female	DD	self-determination theory	intervention	accelerometer
Robertson (2018) [11]	Quantitative	UK	purposive	535	13–20	356 male 179 female	mild to moderate	n/a	longitudinal	questionnaire
Ryan (2018) [37]	Quantitative	Canada	purposive	409	11–23	261 male 148 female	ASD ID	n/a	cross-sectional	questionnaire
Stevens (2018) [38]	Qualitative	UK	purposive	10	16–18	7 male 3 female	mild to moderate	Self-Determination Theory	phenomenology	semi-structured interview
Ptomey (2017) [39]	Mixed method	USA	could not be determined	31	11–21 (13.9 ± 2.7)	16 male 15 female	mild to moderate IDD	n/a	intervention	heart rate monitors, questionnaire, semi-structured interviews
Einarsson (2016) [40]	Quantitative	Iceland	convenience	184 (91ID/93TD)	6–16	could not be determined	mild to severe	n/a	cross-sectional	accelerometers, questionnaire
Pitchford (2016) [41]	Quantitative	USA	convenience	113	2–21	72 male 41 female	DD	n/a	cross-sectional	questionnaire
Queralt (2016) [42]	Quantitative	Spain	convenience	35	15.3 ± 2.7	22 male 13 female	mild to moderate	n/a	cross-sectional descriptive	pedometers
Stanish (2016) [43]	Quantitative	USA	could not be determined	98 (38ID/60TD)	13–21	17/36 male 21/24 female	mild to moderate	social cognitive	cross-sectional	questionnaire
Boddy (2015) [44]	Quantitative	UK	convenience	70	5–15	57 male 13 female	ASD non-ASD	n/a	cross-sectional	accelerometers, quantitative observation (SOCARP)
Eguia (2015) [45]	Quantitative	Philippines	convenience	60	5–14	51 male 9 female	mild to moderate	n/a	cross-sectional	pedometers
Njelesani (2015) [46]	Qualitative	Trinidad and Tobago	purposive	9(parent)	(child) 10–17	(child) 6 male 3 female	moderate to severe DD	occupational perspective	phenomenology	semi-structured interviews, in-depth interviews
Pan (2015) [47]	Quantitative	China (Taiwan)	convenience	80 (40D/40TD)	12–17	30/30 male 10/10 female	21 slight 14 medium ID 3 high ID 2 total ID	n/a	cross-sectional	accelerometer
Downs (2014) [48]	Qualitative	UK	purposive	23 (teachers)	(child) 4–18	(teacher) 9 male 14 femle	ID level could not be determined	n/a	phenomenology	semi-structured focus groups
Downs (2013) [49]	Qualitative	UK	purposive	8	6–21 (16.38 ± 5.04)	3 male 5 female	DS	n/a	phenomenology	semi-structured interview
Shields (2013) [50]	Quantitative	Australia	could not be determined	68	17.9 ± 2.6	30 male 38 female	mild to moderate DS	n/a	intervention (RCT)	accelerometer
Barr (2011) [51]	Qualitative	Australia	purposive	20 (parent)	(child)2–17 (9.9 ± 4.8)	10 female 6 male	DS	n/a	phenomenology	In-depth interview
Temple (2011) [52]	Quantitative	Canada	could not be determined	34 (20ID/14TD)	ID 17.8 ± 1.6 TD 16.4 ± 1.3	10/5 male 10/9 female	mild to moderate	n/a	intervention	questionnaire
Ulrich (2011) [53]	Quantitative	USA	convenience	46	8–15	20 male 26 male	DS	the principles of dynamic systems theory	intervention (RCT)	accelerometers
Lin (2010) [54]	Quantitative	China (Taiwan)	could not be determined	350	16–18	211 male 139 female	mild to profound	n/a	cross-sectional	questionnaire
Pitetti (2009) [55]	Quantitative	USA	purposive	15	8.8 ± 2.2	6 male 9 female	mild	n/a	cross-sectional	heart rate monitor
Sit (2008) [56]	Quantitative	China (Hong Kong)	purposive	80	4–6 grades	54 male 26 female	mild	n/a	cross-sectional	quantitative observation (SOFIT)
Menear (2007) [57]	Qualitative	USA	purposive	21	(child) 3–22	13 male 8 female	DS	n/a	phenomenology	focus group
Faison-Hodge (2004) [58]	Quantitative	USA	convenience	46 (8MR/38TD)	8–11	25 male 21 female	mild MR	social cognitive theory	cross-sectional	quantitative observation (SOFIT), heart rate monitor
Kozub (2003) [59]	Mixed method	USA	could not be determined	7	13–25	4 male 3 female	MR	n/a	cross-sectional	accelerometers, quantitative observation (CPAF), semi-structured interview
Horvat (2001) [60]	Quantitative	USA	purposive	23	6.5–12	could not be determined	mild MR	n/a	cross-sectional	heart rate monitor, accelerometers, quantitative observation
Lorenzi (2000) [61]	Quantitative	USA	purposive	34 (17MR/17TD)	5.5–12	10/10 male 7/7 female	mild MR	n/a	cross-sectional	heart rate monitor, accelerometers, quantitative observation (SOAL)
Sharav (1992) [62]	Quantitative	Canada	convenience	60 (30DS/30TD)	2 –11	could not be determined	DS	n/a	cross-sectional	questionnaire

ASD, autism spectrum disorder; CPAF, the Children’s Physical Activity Form; D, disabilities; DS, down syndrome; DD, developmental disabilities; ID, intellectual disabilities; IDD, intellectual and developmental disabilities; MR, mental retardation; n/a, not applicable; OSRAC-H, the Observational System for Recording Physical Activity in Children-Home; RCT, randomized controlled trial; SOAL, the Scheme for Observing Activity Level; SOCARP, the System for Observing Children’s Activity and Relationships during Play; SOFIT, the System for Observing Fitness Instructional Time; TD, typically developing.

## Data Availability

The data presented in this study are available on request from the authors.
